# Obesity: Its Causes and Treatment

**Published:** 1891-10-31

**Authors:** 


### OBESITY: ITS CAUSES AND TREATMENT.

### II.—Treatment.

A consideration of the causes of obesity, so far as we understand them, will conduce to a rational plan of treatment. The most obvious means is to reduce systematically the consumption of food; but this is a most difficult thing to carry out satisfactorily. No definite quantity can be stated as the normal diet for all men; but it is quite certain that the great majority eat more than is necessary for health. Thirty to forty ounces of chemically dry food are often consumed in the twenty-four hours, whereas fifteen to twenty-five are sufficient for an active man of moderate size. The desire to eat almost invariably exceeds the amount requisite, and in many cases the appetite is so great as to amount to a diseased craving. Habit has much to do with this; it is stated on good authority that the inhabitants of Arctic regions commonly eat twenty pounds weight of food in the day, and Parry, the explorer, narrates the case of an Esquimaux who devoured thirty-five pounds of meat and a number of tallow candles in twenty-four hours. On the other hand, a healthy man can live for nine or ten days without food or water, and *with* water for thirty or forty. No ill effects need result therefore from reduction of diet if it be done gradually and with care.

There are other methods, however, which may be summed up under three heads, (1) increasing the metabolic changes of the tissues, (2) diminishing absorption from the alimentary canal, and (3) diverting the formative (anabolic) processes into other channels than fat formation.

(1) By increasing tissue-change or "metabolism." In 1863 some sensation was caused amongst the corpulent by the publication of a tract by Mr. William Banting on the treatment of obesity by restricting the diet to meat and green vegetables, and avoiding starchy and saccharine food. This pamphlet became famous, and 50,000 copies were soon disposed of. The principle of "Bantingism" is this: Proteid food stimulates tissue change; the diet is almost restricted to meat (green vegetables being chiefly water), and the patient probably eats twice as much animal food as usual. The most successful dieting has been formed on this plan. Oertel, for instance, recommends a decrease in the total amount of food, but an increase in the proportion of nitrogenous material, with active exercise. Ebstein also restricts the consumption of food, but insists on a large proportion of fats, on the theory that by their rapid transference into heat they assist in eliminating other matters. No carbohydrates are allowed.

Mr. Towers Smith narrated some remarkable personal experiences in the *Medical Journal* for October 6th, 1888. He reduced his weight in sixteen days by more than a stone by dieting himself as follows: Breakfast—one pound of rump steak; lunch—one pound of rump steak; dinner—one pound of grilled cod, and one pound of rump steak. One gallon of hot water was drunk during the day; a little whiskey at night, and a few grains of carbonate of potash night and morning. Since then he has treated a large number of patients on these principles with marked success. Anything which increases the circulation also increases the bodily waste; hence tea, coffee, and alcohol in small quantities are useful in conjunction with other methods. Brisk circulation, however, does little good unless combined with free distribution of oxygen. This can best be obtained by pure and bracing air; but may be got, to some extent, by the internal use of permanganate of potash, which readily parts with its oxygen to the tissues.

(2) By diminishing absorption from intestines, &c. Gin and vinegar have an old reputation for reducing fat and keeping their victims thin. This result is brought about by the destructive action of these substances on the digestive walls and juices. They cannot, of course, be employed with safety.

Salts of bromine and iodine, and the alkaline carbonates limit intestinal absorption by causing an osmotic flow into the intestines. This accounts for the successful employment of the waters of Matienbad, and for the fact that the "bladder wrack" (Fucus vesiculosus) has been prescribed with benefit. This sea-weed contains salts of iodine, bromine, and chlorine, and probably produces a copious serous flow from the capillaries of the stomach and intestines. A patent drug called "Anti-fat" has been made from this. Cider and fluids, &c., containing fruit salts act slightly in the same way.

(3) By diverting the formative processes into other channels. This may be brought about by proper exercise of the muscles of the limbs and heart, and by using the nervous system. When these are at work the ill-understood forces which govern metabolic activity are taken away from the useless fat cells and supplied to the nervous and muscular system. To ensure success, however, exercise must be methodical, and must be supplemented by other means. It is important that the heart, and, therefore, the circulation, should be strengthened by work sufficiently laborious to cause quickening of the pulsations, as in Oertel's plan of walking up hills, &c.

We may, in conclusion, point out that probably a combination of the three methods indicated, carried out with great firmness and persistence, will be necessary in order to obtain good results.

